# Rational belief, epistemic possibility, and the a priori

**DOI:** 10.1007/s44204-024-00137-y

**Published:** 2024-01-25

**Authors:** Claire Field

**Affiliations:** COGITO, University of Glasgow, Glasgow, UK

**Keywords:** Rational belief, Epistemic possibility, A priori

## Abstract

In this paper, I discuss Whiting’s (2021) account of rational belief and discuss some unresolved issues arising from its reliance on epistemic possibility and, by extension, perspective-relative aprioricity.

## Rationality and rightness

On Whiting’s view (2021), it is epistemically right to believe what is true and only what is true. However, in line with most views of epistemic rationality, it is often rational to believe what is false. Whiting characterises rational belief in the following way:

Rational belief: Necessarily, it is rational for a person to believe a proposition if and only if there is a subjective reason for them to do so. (p. 179)

He gives the following definition of a subjective epistemic reason (p. 175):

What appears to a person to be the case, P, is a subjective-justifying epistemic reason for them to believe a proposition if and only if:

(i) in every nearby *epistemically* possible world in which P obtains that proposition is true.
(ii) they have the general ability to believe the proposition for the reason P.

Whiting endorses a modal account of reasons, according to which whether there is a reason R depends on whether R obtains in nearby possible worlds. While objective-justifying epistemic reasons depend on how things are in nearby *metaphysically* possible worlds, subjective-justifying reasons depend on how things are in nearby *epistemically* possible worlds. While the objective epistemic reasons determine what is known, the subjective epistemic reasons determine what can be believed rationally. Epistemically possible worlds are worlds a person cannot rule out *a priori*. They are ‘maximal possibilities that are *a priori* coherent for a person’ (p. 174). To take Whiting’s example, Miyuki sees that the listings state that her favourite film is showing. It is rational for Miyuki to believe that her favourite film is showing, because relative to Miyuki’s epistemic perspective (her background beliefs, memories, and so on), the possibility that the film is not showing is a remote one—in all the close epistemically possible worlds, the film is showing. So, this means that relative to those epistemically possible worlds, she can rule out *a priori* the possibility that the film is not showing. This is true even if, by some strange quirk such as a misprint, the film is not showing.

On this account, what is rational for someone to believe depends on what is epistemically possible, which in turn is determined by what that person can rule out *a priori*. However, determining what is *a priori* for someone is not always straightforward. How we understand this has direct and important implications for how a modal account of rational belief, like Whiting’s, understands rational belief.

## What is a priori?

P is epistemically possible for S when she cannot rule out S *a priori*, given her total set of knowledge, evidence, background beliefs, and so on. In other words, P cannot be ruled out *a priori* if it is consistent with S’s current epistemic state.

However, what someone can rule out *a priori* also seems to depend on her epistemic abilities. For example, a beginning logic student will be able to rule out fewer logical falsehoods than an expert, in virtue of their inferior ability to perform *a priori* logical reasoning. In general, we cannot assume that all agents have the same epistemic abilities. This means that what is epistemically possible will vary between agents, and so what subjective epistemic reasons they have, and therefore what it is rational for them to believe, will also vary. Indeed, Whiting embraces this. For him, epistemic possibility is subject-dependent: ‘[w]hether a world is epistemically possible for a person depends on their cognitive capacities’ (90). For example, there are some mathematical possibilities that I cannot rule out a priori, but that someone with greater ‘mathematical insight or ability’ (90) would be able to rule out. So, different things would be rational for each of us to believe. This means that Whiting rejects the following Bayesian View of what is a priori:

- Bayesian View: All logical and conceptual truths are *a priori* for all agents.

Ideal agents have credence 1 in all logical truths and know these truths a priori. Bayesian Views say that to have anything short of maximal confidence in the truths of logic is to fail to meet the ideals of rationality. This is obviously very demanding. One consequence of adopting this understanding of a priority in conjunction with Whiting’s modal account of rational belief would be that false rational belief about logical or conceptual propositions would not be possible. All logical and conceptual truths would be true in all epistemically possible worlds, and so all agents would always have subjective epistemic reason to believe all a priori truths. Rational false belief about a priori truths would be impossible. It is thus understandable that Whiting rejects this view of the a priori. There are many plausible examples of agents incorrectly doing very complex logical reasoning and coming to believe something false about logic. Frege’s acceptance, and then later rejection, of Basic Law V is one example of this. Instead, on his view, cognitive capacities affect what is a priori for someone. But, how, exactly? Various views on this are possible. We might be tempted by something like the following view:

- Restricted View: All sufficiently obvious logical and conceptual truths are a priori for all agents.

The Restricted View restricts the set of propositions that are a priori to a subset of the total logical and conceptual truths—those that are sufficiently obvious. Which propositions are ‘sufficiently obvious’ in the relevant sense? Following Harman (1986), we might understand this to include some specific set of propositions, perhaps very basic logical truths, and the immediate logical implications of one’s current beliefs. This would be to take an objective view of what is a priori—like the Bayesian View, the Restricted View would then say that there is a unique set of propositions that is a priori for all agents, though a much smaller set than the Bayesian View assumes.

Of course, what is obvious could also vary between individuals based on their epistemic capacities. What will seem obvious to an expert logician is very different to what would seem obvious to a novice. This ambiguity in whether obvious is to be read objectively (as Harman does) or subjectively (i.e. as what ‘seems’ obvious to someone) suggests the following disambiguation:

- Restricted View 1: A subset of the total logical and conceptual truths, those that meet some objective threshold of simplicity, are a priori for all agents.

- Restricted View 2: P is a priori for S if it seems sufficiently obvious to her, given her epistemic capacities.

Restricted View 2 generates a reading of what is a priori according to which this is highly dependent on the individual’s epistemic capacities, for example, her capacities in logical and conceptual reasoning. This allows us to avoid saying that novices cannot rationally believe falsehoods, because not all logical truths need be thought of as epistemically possible for all agents. However, we might wonder whether obviousness provides the right kind of restriction—perhaps some propositions could be a priori for someone, due to the nature of her epistemic capacities, without seeming obvious to her.^1^

We also might wonder whether false propositions can also be a priori for someone. Certainly, if epistemic possibility is subject-dependent, then there are some false propositions that some subjects cannot rule out a priori, which makes it rational for them to believe them. For example, Whiting says: ‘What is a priori for one person might differ from what is a priori for another […] what is a priori for a person depends on their capacities for reasoning, intuition, and the like. So, while it is rational for Sophia to believe that 1+1=2, it might not be rational for her to believe that 0.999...≠1’ (p.188). This may not be *quite* the same thing as saying that false propositions are a priori, but it generates the result that false propositions about traditionally a priori matters can be believed rationally. This represents an interesting departure from previous attempts to Whiting to reduce the demandingness of Bayesian commitments to logical omniscience, which have typically seen the difference between what is a priori for different individuals in terms of restrictions on the Bayesian requirement to believe *all* logical truths.

This subject-dependent account of epistemic possibility raises further questions about epistemic abilities.

## How what is a priori affects rational belief

However, if what is a priori depends on our epistemic abilities, and these in turn affect which subjective epistemic reasons we have and therefore what we are rational to believe, we might wonder what, exactly, affects these abilities. For example, do all rational agents possess the same epistemic abilities? Are they affected by psychological features such as processing speed, intelligence, and linguistic skills? Are they affected by our evidence? This matters for Whiting’s account of rational belief, because if the analysis of rational belief depends on how we understand what is a priori for someone and what is a priori for someone depends on their epistemic abilities, then how we understand epistemic abilities will affect what they can rationally believe. This is particularly relevant in cases of false, and apparently rational, beliefs about a priori matters such as logic and philosophy. Consider the following two cases:

Case 1: Heloise is considering a complex logical tautology T. T is very complex and would require a great deal of time and logical study to prove. Heloise has only basic logic skills.

Case 2: Magnus is an expert logician and has studied T for some time. He has completed a proof of T. However, right now he is drunk and has forgotten how the proof goes.

Neither Heloise nor Magnus currently has the ability to believe T with a priori justification. However, there is an intuitive sense in which T is a priori for Magnus, at least when he is not drunk, in a way it is not for Heloise. However, if a priori abilities are to ground a theory of rational belief, more should be said about which psychological factors determine epistemic abilities, particularly a priori abilities.

One way to understand epistemic abilities is as relative to distinct contexts. Different ability attributions seem appropriate given different contextual framings. For example, there is a sense in which, in virtue of being a human of normal cognitive capacity, I have the ability to speak Finnish. That is, if I were to take Finnish lessons and spend time acquiring the language. I am the sort of being who could, if she tried, learn to speak Finnish. However, I cannot *now* speak Finnish. Relative to the context of what I have the ability to do *now*, I do not have the ability to speak Finnish. Relative to yet another context, consider a native Finnish speaker who has taken a vow of silence. There is a sense in which she can speak Finnish, but to do so would mean breaking her vow. It is nevertheless felicitous to imagine her saying (or writing, perhaps) ‘It would be impossible for me to speak Finnish now, I have taken a vow not to’. Following Mele (2003), we might understand these different contexts in terms of general and specific abilities. I have the general ability to speak Finnish, but not the specific ability. We might also say that the native Finnish speaker has the general ability but lacks the specific ability, though for different reasons.^2^ Indeed, Whiting makes use of this distinction in defining what it is to possess a reason for action: to possess a reason for action is to have the specific, and not merely the general, ability to act in light of it (see §5.2).

Applying this to the a priori cases, we could say that both agents have the general ability to believe the proposition, but not the specific ability. Or, perhaps only Magnus has the general ability and neither have the specific ability (because Magnus is drunk and Heloise lacks the general ability). This invites a further question: Which of these contexts are context for assessing the abilities that determine what is a priori for someone? This is important because it determines what can and cannot be believed rationally. Consider the following case^3^:

Logic 101: Sam is a typical undergraduate student with ordinary logical capabilities enrolled in an introductory logic class. Unluckily, her professor is a dialetheist who intends to set his students on the ‘right track’ by exposing them to all the best arguments in favour of dialetheism and all the worst arguments against it. By studying the arguments, familiarising herself with dialetheic proof theory, and generally immersing herself in dialetheism, Sam comes to hold various false logical beliefs.^4^

Is Sam rational to hold these false logical beliefs, or not? On Whiting’s view, this depends on what is epistemically possible for her and, therefore, what is and is not ruled out a priori, and this in turn depends on Sam’s epistemic abilities. Determining the right way to understand these things is difficult to do in a theory-neutral way.

For example, in defining what is a priori for Sam, we might consider only the very general abilities that agents like her have. We might think that all rational agents have general abilities to believe basic logical truths. If so, then the logical truths are epistemically possible for her, despite her indoctrination into dialetheism. Adopting this view would put us in agreement with Bayesians and others who think we always have a priori propositional justification for truths of logic (see Kitcher, 1980, 2000; Ichikawa & Jarvis, 2013; Titelbaum, 2015). Whiting, of course, rejects this view. Indeed, perhaps the more appropriate ability attribution here is one that takes into account her evidential situation. Perhaps, in virtue of being a rational agent with lots of evidence supporting false logical beliefs, she does not have the ability to believe anything other than what her evidence supports (or appears to support^5^). This would put us in agreement with those who have thought we can be a priori justified in believing what is false as well as what is true (see Summerfield, 1991; Casullo, 2003; De Toffoli, 2021). Indeed, Whiting sometimes seems to suggest this reading of a priori abilities. For example, in outlining epistemic possibility for the first time, he says that ‘there are mathematical propositions that I am unable to rule out a priori but that a person with greater mathematical insight or ability can rule out a priori. So, there are mathematical propositions – hence, worlds in which those propositions hold – that are epistemically possible for me but not for them’ (p. 90).

To apply this to Logic 101, we need to know whether to think that misleading evidence can affect our logical ‘insight or ability’. It certainly might affect what Sam finds plausible. So, in this sense it might affect what she can rule out a priori. Though, equally, we might think that logical ability (and a priori abilities more generally) cannot be so easily affected by receiving misleading evidence.

Suppose that evidence can affect ability and make it such that the false beliefs about logic are epistemically possible for her, because she can no longer rule them out. Does this also make her rational to believe them? Does she have subjective epistemic reasons to believe them?

Again, this is tricky. Whiting’s view says that S is rational to believe P when there is a subjective justifying reason to do so. There is a subjective justifying reason R for P when R is *true* in nearby epistemically possible worlds, and S has the ability to believe P for R. Some of the potential justifying reasons will also be logical falsehoods. Could these logical falsehoods be true in nearby epistemically possible worlds? If epistemic possibility is defined by what is a priori, and what is a priori is defined by what we cannot rule out, and we think Sam’s misleading evidence supporting dialetheism means he cannot rule out the false truths, then it seems we must think they are true in nearby epistemically possible worlds—that is just what epistemic possibility is.

However, we might think this risks distorting the concept of the a priori too far. A priori reasoning is traditionally distinguished from a posteriori reasoning because on the assumption that there is something distinctive about its independence from experience. This idea is what pushes many of its proponents to postulate basic a priori competencies that are unaffected by misleading evidence of the sort that Sam acquires in Logic 101.^6^ However, if something like having misleading evidence for an opposing view could alter what counts as a priori for an agent, then we might worry that this is really a way of denying the existence of the a priori. A priori justification, in this case, is not really independent of experience because it can be affected by experiences we have—like gaining evidence.

This worry might push us to resist an account of rational belief that relies on a notion of a priority.

## False beliefs about philosophy

Another interesting problem arises when we consider false normative beliefs about epistemology, which are also presumably a priori.

Consider Whiting’s preferred view of epistemic rightness:

TRUTH: Necessarily, it is right for a person to believe a proposition if and only if that proposition is true. (p. 175)

Presumably, it is possible to be mistaken about this. Someone might endorse one of the following, assumed false, theories of epistemic rightness:

EVIDENCE: Necessarily, it is right for a person to believe a proposition if and only if that proposition is supported by her evidence.

KNOWLEDGE: Necessarily, it is right for a person to believe a proposition if and only if they know that proposition.

These alternative views on epistemic rightness are held by some philosophers.^7^ Can they be held *rationally*? Some have disputed this, because if false beliefs about what rationality requires are possible, this leads to inconsistency within rationality—at least, if we think that epistemic rationality also requires level-coherence.^8^ That is, if we think epistemic rationality requires wide-scope coherence between our normative epistemic beliefs and the rest of our beliefs. However, since Whiting’s account of rational belief depends on what can be ruled out a priori, there are presumably situations in which someone would not be able to rule out EVIDENCE or KNOWLEDGE a priori, either because they lack the philosophical competence, or because they have misleading evidence supporting or appearing to support these views.^9^

Assuming this is right, how should this affect the rest of their beliefs? A natural thought is that one’s beliefs about what is epistemically required ought to affect the rest of one’s beliefs. However, Whiting’s account also permits level-incoherence. He says that the following principle is false (p.186):

R<->RR Necessarily, it is rational for a person to believe a proposition, iff it is rational for them to believe that it is rational for them to believe that proposition.

This allows for possible cases of epistemic inadvertent virtue: cases in which an agent rationally believes something right, while believing that it is neither rational nor right.

However, we might wonder whether such beliefs could really be rational, given Whiting’s account of rational belief. Recall that S rationally believes P when she has a subjective epistemic reason to believe P. So, could someone who rationally believe a false view, such as EVIDENCE, have a subjective epistemic reason to believe something that was in fact right, but prohibited by EVIDENCE?

In the closest epistemically possible worlds where EVIDENCE is true, it is epistemically right to believe what one’s evidence supports (even when this means not believing the truth).

In those worlds, there will be some truths that are prohibited by EVIDENCE but required by TRUTH (which is, in fact, the true view of epistemic rightness). The fact that a proposition is prohibited at the closest epistemically possible worlds seems to generate a subjective epistemic reason to not believe those propositions. So, someone who rationally believes EVIDENCE would be rational also to not believe things it prohibits, even when those things are in fact epistemically right. However, this seems to suggest that there cannot be cases of inadvertent epistemic virtue after all. It also seems to suggest that it is not always rational to believe what is right.

Is this is the right result? On the one hand, we might think it is. Being in the grip of a false view can change what it is rational for you to believe, sometimes for the worse. However, we also might then wonder if R<->RR has really been rejected? At least, when the agent has false beliefs about what rationality requires, this principle seems to hold up.

## Summary

In summary, Whiting offers a novel and interesting account of rational belief. However, its reliance on what is a priori raises some tricky questions on how to understand rational false beliefs about a priori propositions. Depending on how we interpret it, it either has admirable flexibility in what can be rationally believed, or risks distorting what it is to be a priori justified beyond plausibility. Either way, this is a valuable and welcome contribution to the literature on rational belief.^10^

## Data Availability

There is no data associated with this manuscript.

## Footnotes

1 Thanks to Whiting for pointing this out.

2 Though there is also something odd about this, nothing is physically stopping them from speaking Finnish in the way that my lack of study is.

3 See Field (2019, 2020, 2021) for further discussion of this case.

4 For the details of dialetheism, see Priest (1979, 1986, 2006).

5 I include this qualification because some might dispute that her evidence really supports the false logical beliefs. Even so, this needs not affect the ability we attribute to Sam in this case. What one’s evidence appears to support is also, plausibly, relevant to our a priori epistemic abilities.

6 Though they say that such evidence might have a ‘disabling’ affect on Sam’s default a priori propositional justification (Smithies, 2015). Or, as Ichikawa and Jarvis put it, it could cause ‘performance errors’, but without affecting basic a priori competence. Importantly, it does not *change* her a priori reasoning competence.

7 For EVIDENCE, see Feldman and Conee (1985); for KNOWLEDGE, see Williamson (2000).

8 See Littlejohn (2018) and Titelbaum (2015). See Field (2019, 2020, 2021) for arguments against this.

9 Indeed, Whiting notes (p. 126) that it would be ‘coherent’ (where read in context this seems equivalent to ‘rational’) to believe alternative accounts of epistemic rightness.

10 I would like to thank Daniel Whiting, Davide Fassio, and the other participants of this symposium (in particular, the anonymous participant who reviewed the paper) for their helpful comments on earlier draughts of this paper. I am grateful to Daniel Whiting and Davide Fassio for the opportunity to think in depth about these issues and for organising the associated workshop through which these ideas were developed. This research has received funding from the European Research Council (ERC) under the European Union’s Horizon 2020 research and innovation programme (KnowledgeLab: Knowledge-First Social Epistemology project, grant agreement no. 948356, PI Mona Simion).
